# SphK1‐driven autophagy potentiates focal adhesion paxillin‐mediated metastasis in colorectal cancer

**DOI:** 10.1002/cam4.4129

**Published:** 2021-07-16

**Authors:** Jiang‐Ni Wu, Lan Lin, Shi‐Bo Luo, Xin‐Ze Qiu, Li‐Ye Zhu, Da Chen, Er‐Dan Wei, Zhen‐Hua Fu, Meng‐Bin Qin, Zhi‐Hai Liang, Jie‐An Huang, Shi‐Quan Liu

**Affiliations:** ^1^ Department of Gastroenterology The Second Affiliated Hospital of Guangxi Medical University Nanning Guangxi P.R. China; ^2^ Department of Gastroenterology The First Affiliated Hospital of Guangxi Medical University Nanning Guangxi P.R. China

**Keywords:** autophagy, colorectal cancer, metastases, paxillin, sphingosine kinase 1

## Abstract

Invasion and metastasis are the main causes of colorectal cancer (CRC)‐related death. Accumulating evidence suggested that sphingosine kinase 1 (SphK1) promoted the metastasis of CRC and autophagy played an important role in SphK1 promoting the metastasis of malignancy. However, the mechanism by which SphK1‐driven autophagy promotes invasion and metastasis in CRC remains to be clarified. In the present study, immunohistochemical detection showed the expression of SphK1 and paxillin was higher in human CRC tissues than those of normal colorectal mucosal tissues, they were both associated with TNM staging, lymphatic, and distance metastasis. In addition, study of in situ tumor transplantation model in nude mice showed that the suppression of SphK1 inhibited the growth of colonic orthotopic implantation tumors and the expression of paxillin, p‐paxillin, LC3 in the tumor. So, SphK1 may promote CRC metastasis via inducing the expression of paxillin expression and its phosphorylation, in vivo. Furthermore, results of CCK8 assay, transwell and wound healing assays showed that SphK1 promoted the viability, invasion, and metastasis of CRC cells. Transmission electron microscopy detection showed that SphK1 is the key factor in autophagy induction in CRC cells. Moreover, western blot examination indicated that the expression of LC3Ⅱ/Ⅰ, paxillin, p‐paxillin, MMP‐2, and vimentin was enhanced in SphK1‐overexpressed CRC cells and suppressed in SphK1 knockdown CRC cells, meanwhile, the expression of E‐cadherin was suppressed in SphK1‐overexpressed CRC cells and enhanced in SphK1 knockdown CRC cells. Suppression of autophagy by 3MA reversed the expression of paxillin and its phosphorylation in SphK1‐overexpressed CRC cells, indicated that SphK1‐driven autophagy induced the expression of paxillin and its phosphorylation in CRC cells. Together, these findings reveal that SphK1‐driven autophagy may promote the invasion and metastasis of CRC via promoting the expression of focal adhesion paxillin and its phosphorylation.

## Graphical abstract

SphK1‐driven autophagy may promote the invasion and metastasis of CRC via promoting of the expression of focal adhesion paxillin and its phosphorylation.

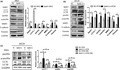

## 1 INTRODUCTION

Colorectal cancer (CRC) ranks third for incidence and second for mortality all over the world.^1^ Invasion and metastasis are the main causes of cancer‐related death. Thus, identification of the mechanism of CRC invasion and metastasis may provide new targets for the therapy of CRC.

Sphingosine kinase 1 (SphK1), a regulator of sphingolipid metabolites, involves in the process of cancer including proliferation, invasion, and metastasis and it is associated with the prognosis of various cancer.^2^ Recently, accumulating evidence suggested that autophagy played an important role in SphK1 promoting the development and metastasis of malignancy.^3^ It is reported that SphK1‐driven autophagy of human peritoneal mesothelial cells accelerated gastric cancer peritoneal dissemination,^4^ and microRNA‐506‐3p suppresses autophagy and cell invasive capability in osteosarcoma cells by targeting SphK1.^5^ Furthermore, SphK1 accelerated the lysosomal degradation of CDH1 to induce EMT, which depended on TRAF2‐mediated macroautophagy/autophagy activation.^6^ In CRC cells, previous study found that the autophagy can be induced by SphK1.^7^ So, SphK1‐driven autophagy may potentiate the metastasis in CRC, however, the precise mechanism is still uncovered.

Previous study showed that SphK1 promoted the metastasis of CRC via the focal adhesion kinase (FAK) singling pathway.^8^ FAK is a key regulator of focal adhesions (FA), which mediates the adhesion between cells and extracellular matrix and participates in cell migration and signal transduction.^9^ The assembly of FA can produce migration forces that are conducive to cell movement and anchored on the matrix, and FA disassembly can further promote cell migration.^10^ As a major scaffolding protein of FA, paxillin plays critical role in the assembly and disassembly of FA (FA turnover).^11^ Paxillin contains five leucine‐aspartic acid (LD) motifs at its N‐terminal and four Lin11, Isl‐1, and Mec‐3 (LIM) domains at its C‐terminal, LD motifs mainly control most of its signal conduction activity, and LIM domains mainly mediate the protein interaction.^12^, ^13^ It has been proved that paxillin involves in the regulation of the cytoskeleton, tumor invasion, and metastases.^14^ Study showed paxillin was associated with lymph node metastasis in prostate cancer, and downregulated paxillin suppressed the invasion of colon cancer cells.^13^, ^15^ Recent studies have shown that paxillin phosphorylation promotes the adhesion between cancer cells and matrix, inhibits the adhesion between cells, and promotes pseudopodia formation of cancer cells, thus promoting invasion and metastasis of cancer cells.^16^ In recent years, FA turnover and autophagy came into view. A study had shown that selective autophagy promotes migration through enabling FA turnover.^17^ Hence, SphK1‐driven autophagy may potentiate the metastasis in CRC via regulating the turnover of FA.

Therefore, the aim of this study was to investigate the role of SphK1 on autophagy, focal adhesion paxillin, and the metastatic capabilities of cancer cells in vitro and in vivo. Furthermore, the role of SphK1‐driven autophagy activation and its influence on focal adhesion paxillin and the metastatic capabilities of cancer cells were examined.

## 2 MATERIALS AND METHODS

### 2.1 Patients and tissue samples

The study included 91 tumor tissues and 26 normal tissues. These 91 CRC patients aged from 25 to 83 years. Based on the National Comprehensive Cancer Network classification,^18^ there were 13 patients at stage I, 33 patients at stage II, 26 patients at stage III, and 19 patients at stage IV of the disease.

### 2.2 Reagents and antibodies

CCK8 kit (FD3788) was purchased from Fude biological (Hangzhou, China). Anti‐LC3I/II (M186‐3) was purchased from MBL (Boston, MA, USA), anti‐SphK1 (ab71700) was purchased from Abcam (Cambridge, UK), anti‐human E‐cadherin (3195) and vimentin (5741) were purchased from Cell Signaling Technology (Beverly, MA, USA), anti‐paxillin (10029‐1‐lg) was purchased from Proteintech (Wuhan, China), anti‐phospho‐paxillin (bs‐5548R) was purchased from Bioss (Beijing, China), anti‐MMP2 (A11144) was purchased from Abclonal (Wuhan, China), and GAPDH (21612) was purchased from SAB (California, USA). 3‐Methyladenine (3MA, T1879) was purchased from TargetMol (Shanghai, China).

### 2.3 Immunohistochemistry detection (IHC)

The tumor tissues were fixed in formalin and embedded by paraffin, then the paraffin samples were sectioned. The slides were roasted, dewaxed, hydrated, and rinsed. Then the tumor sections were put in citric acid for antigen retrieval and the endogenous peroxidase activity was inactivated by 3% H_2_O_2_. Subsequently, the tumor sections were blocked in normal goat serum at room temperature for 1 h, successively incubated with primary antibodies at 4°C overnight, secondary antibody and horseradish peroxidase‐conjugated. More details about DAB coloration, counterstain, and hydrochloric acid alcohol differentiation were performed according to the guidance of SP immunohistochemical kit (ZSGB‐BIO, SP‐9000, Beijing, China). The protein expression analysis of half quantitative was observed by five random fields of each slide. The staining intensity and the percentage of positive cells jointly define. The staining intensity was scored as: 0; achromatic, 1; light yellow, 2; yellow, and 3; brown. And the result of each percentage of positively stained: cells <5% was scored as 0, 5%–25% scored as 1, 26%–50% scored as 2, and >50% scored as 3. The two scores were multiplied for the final score: 0–2 score: (−), 3–4 score: (+), and a score of 5 or above: (++).

### 2.4 Cell culture and transfection

The siRNA sequence (CCC AAA CTA CTT CTG GAT GGT) targeting SphK1 gene is connected to gv248 vector which derived from Genechem and packaging plasmids were used to co‐infect HEK293 as knockdown lentivirus particles. The insignificant siRNA sequence was constructed on gv248 vector and co‐transfected with helper plasmid to package HK293 cells as control lentivirus particles. The CRC RKO cells were inoculated with lentivirus at the optimal MOI which is above 80% of infected cells. Lentiviral vector encoding human SphK1 (Lenti‐SPHK1‐IRES‐EGFP; 2 × 10^9^ TU/ml) was introduced into HT29 cells via lentiviral transfection. HT29 cells were additionally transfected with the blank vector (Lenti‐EGFP; 5 × 10^8^TU/ml). The POLOdeliverer™ 3000 Transfection Reagent was purchased from Genechem Co., Ltd (Shanghai, China). Stably transfected cells were selected for further experiments. All cells were maintained in DMED medium (Gibco, USA) supplemented with 10% fetal bovine serum (FBS; ExCell Bio, China) and 1% penicillin–streptomycin mixture (ExCell Bio, China) at 37°C, 5% CO_2_.

### 2.5 RNA isolation and reverse transcription‑quantitative (RT‐q) PCR analysis

Total cellular RNA isolation was performed from cells using the Total RNA extraction kit and reversely transcribed into cDNA using a reverse transcription kit (both From Applied Biological Materials Inc, Canada). qPCR was performed using the Power SYBR Green Master Mix (Genstar, Beijing, China) on an RT‑qPCR reaction was run on a StepOne RT‐PCR system (Applied Biosystems; Thermo Fisher Scientific, Inc.). The primers used were SphK1 forward 5'‐GGC TTC ATT GCT GAT GTG GA‐3’, and reverse 5'‐AGG AAG GTG CCC AGA GTG AA‐3’; and GAPDH forward, 5'‐GCA CCG CAA GGC TGA GAA C‐3’ and reverse, 5'‐TGG TGA AGA CGC CAG TGG A‐3’; which were all purchased from Sangon Biotech Co., Ltd. (Shanghai, China). The cycling parameters were as follows: Denaturing at 95°C for 2 min, 40 cycles of denaturing at 95°C for 15 s, primer annealing at 60°C for 15 s and extension temperature at 95°C for 15 s, and final extension at 60°C for 15 s and final denaturing at 95°C for 15 s. Results were quantified using the 2^−ΔΔCq^ method.

### 2.6 Cell viability assay

The 96‐well pate was filled with cells at a density of 5 × 10^3^ cells per well overnight. After attachment, cell viability was examined with 10 μl of 5 mg/ml CCK8 solution added to each well for 1–4 h following the kit's manual.

### 2.7 Western blot

Cells were homogenized and lysed in RIPA buffer (Beyotime, China) supplemented with proteinase inhibitors (Solarbio, China) and phosphatase inhibitors (NCM Biotech, China). Protein quantification was performed with the BCA assay (Beyotime, China) and equal amounts of proteins (30 μg) were separated and run on 10%–15% SDS‐PAGE gel following electrophoresis at 80 V for 2.5 h, then transferred onto the polyvinylidene fluoride (PVDF; Millipore, USA) membranes at 250 mA for 2.5 h. After blocked with 5% milk in 1 × TBST for 1 h, membranes were incubated with antibodies overnight at 4°C with the primary antibodies: SphK1 (1:1000, Abcam), LC3 (1:1,000, MBL), paxillin (1:1000, Proteintech), MMP2 (1:1000, Abclonal), p‐paxillin (1:800, Bioss), E‐cadherin (1:1000, CST), vimentin (1:1000, CST), and secondary antibody at room temperature for 1 h in lucifugal environment. GAPDH (1:10000, SAB) was used as the loading control. Images and band intensity were quantitated with Odyssey CLx Infrared Imaging System (LI‐COR Biosciences, Lincoln, NE, USA).

### 2.8 Wound healing and transwell migration assays

For the wound healing assay, cells were scratched with a tip with 90% confluence rate (triplicates per group) and washed with PBS, then cultured in serum‐free medium for 48h. Photographs of random fields were captured for analysis under a microscope (Nikon Corporation).

For transwell migration, 2.5 × 10^5^/ml cells suspended in serum‐free medium were added to the upper chambers of transwell filter (8 μm pore size; Corning), and 700 μL 10% FBS was added to the lower chambers (triplicates per group). After 48 h co‐culture, the cells that had migrated to the lower surface of membrane were fixed in 4% paraformaldehyde and Giemsa‐stained, the migrated cells were then counted under a microscope (Nikon Corporation). Photographs of random fields were captured for analysis under a microscope (Nikon Corporation).

### 2.9 Transmission electron microscope

SphK1(‐)‐RKO, NC‐RKO, SphK1(+)‐HT29, and NC‐HT29 cells were fixed with 2.5% glutaraldehyde, stored at 4°C overnight, the cell pellet was embed with 1% agarose, and postfixed with 1% OsO4 for 2 h. After dehydration through ethanol, infiltrate with proportional of acetone and EMbed 812, embed by baking in 60°C. Subsequently, they were cut into ultrathin sections. Then sections were stained with uranyl acetate and lead citrate. Finally, ultrathin sections were examined through a transmission electron microscope.

### 2.10 Animal experiments

Nude mice (five per group, divided into SphK1(‐)‐RKO and NC‐RKO group, male and female unlimited) were provided by Guangxi Medical University. Subcutaneous xenografts were, respectively, established by injection of 1 × 10^7^ CRC cells including SphK1(‐)‐RKO and NC‐RKO in dorsal surfaces animals. Once the tumors grown to approximately 1 cm^3^, they were cut into 1 mm^3^ pieces then one of that orthotopically implanted into the serous layer of the colon with medical OB glue. After the glue coagulated, the abdominal wall was seamed by absorbable suture. During surgical procedures, mice were anesthetized with 0.5% pentobarbital sodium intraperitoneal injection. Each mouse in the two different groups was observed at least 5 weeks before they were sacrificed. The primary tumors were measured and snap frozen in liquid nitrogen for further experiments.

### 2.11 Statistical analysis

The mean ± SD are used for all results. All experiments were repeated at least three times. Student's *t*‐test or ANOVA test was used to analyze the data by SPSS 22.0 software (SPSS, Inc., Chicago, IL, USA). The frequencies in the different groups were evaluated using the Fisher's exact test. *p* < 0.05 was considered to indicate a statistically significant difference.

## 3 RESULTS

### 3.1 Expression and clinical feature of SphK1and paxillin in human CRC tissues

**FIGURE 1:**
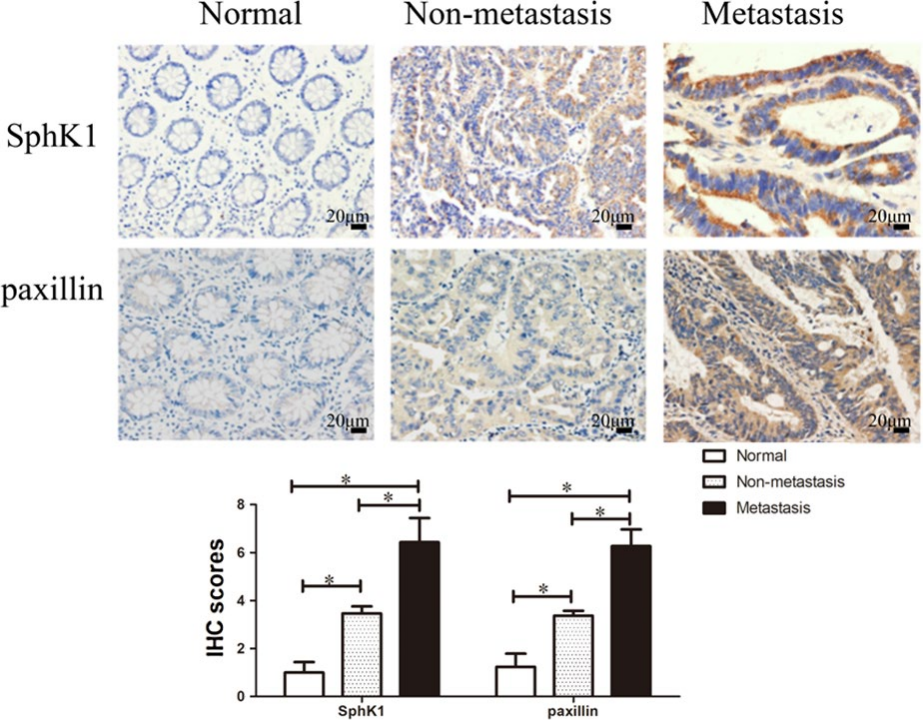
Aberrant expression of SphK1 and paxillin in CRC. Immunohistochemical staining of SphK1 and paxillin in CRC and normal colorectal mucosal tissues (magnification, ×400, *p < 0.05 vs. normal or non‐metastasis group). Data are shown as the mean ± SD of three replicates.

Expression of SphK1 and paxillin in CRC tissues was significantly stronger than those in the normal colorectal mucosal tissues, meanwhile, the expression of SphK1 and paxillin in CRC tissues with metastasis was higher than those in CRC tissues without metastasis (Figure 1). The expression levels of SphK1 and paxillin were significantly relevant to tumor pathological stage, presence of lymphatic metastasis, and development of distant metastasis (Table 1), moreover, the expression of paxillin is closely correlated with the expression of SphK1 in CRC tissues (Table 2).

**TABLE 1:** SphK1 and paxillin expression in CRC tissues and clinical significance of patients with CRC

Pathologic feature	N	SphK1			Paxillin		
		−	+	*p*	−	+	*p*
Infiltration depth				0.453			0.963
Mucosa and superficial muscular layer	14	6	8		5	9	
Deep muscular layer and below	77	22	55		28	49	
Sex				0.881			0.386
Male	55	16	39		18	37	
Female	36	11	25		15	21	
TNM staging				0.008			0.000
Ⅰ stage	13	7	6		11	2	
Ⅱ stage	33	14	19		12	21	
Ⅲ stage	26	6	20		7	19	
Ⅴ stage	19	1	18		3	16	
Lymphatic metastasis				0.01			0.01
−	50	21	29		24	26	
+	41	7	34		9	32	
Distant metastasis				0.007			0.037
−	72	27	45		30	42	
+	19	1	18		3	16	

**TABLE 2:** Correlation between the expression of SphK1 and paxillin in CRC

Expression	Paxillin				
	−	+	++	Correlation	*p*
SphK1				0.506	0.000
−	20	6	2		
+	8	19	8		
++	5	7	16		

### 3.2 Knockdown and overexpression of SphK1 in CRC cells

**FIGURE 2:**
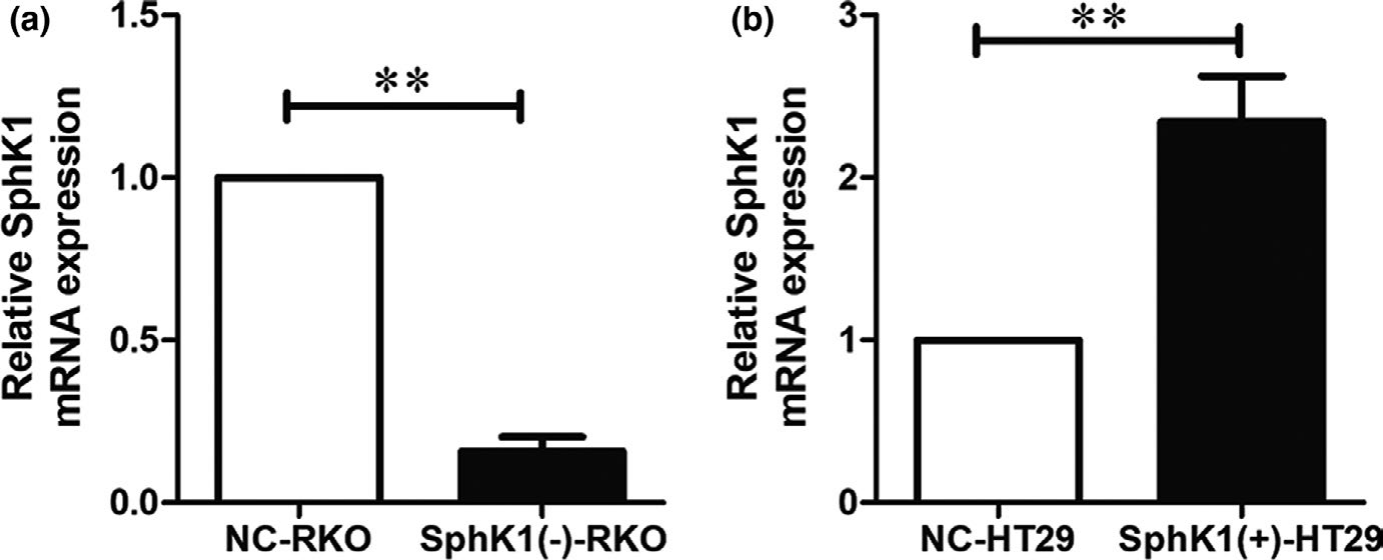
SphK1 was successfully constructed in CRC cells. qRT‐PCR revealing SphK1 mRNA expression in RKO and HT29 cells. The relative expression of SphK1 was quantified via normalization to GAPDH. (**p < 0.01 vs. NC‐RKO or NC‐HT29 group). Data are shown as the mean ± SD of three replicates.

RKO cells were transfected with Sphk1 siRNA lentivirus particles and HT29 cells were used for stable transfection of the SphK1 overexpressing vector. The expression of Sphk1 was examined by RT‐qPCR. Compared with NC group, SphK1 mRNA were successfully suppressed in the transfected RKO cells. And it was significantly increased in the transfected HT29 cells (Figure 2).

### 3.3 SphK1 affected the growth of CRC cells

**FIGURE 3:**
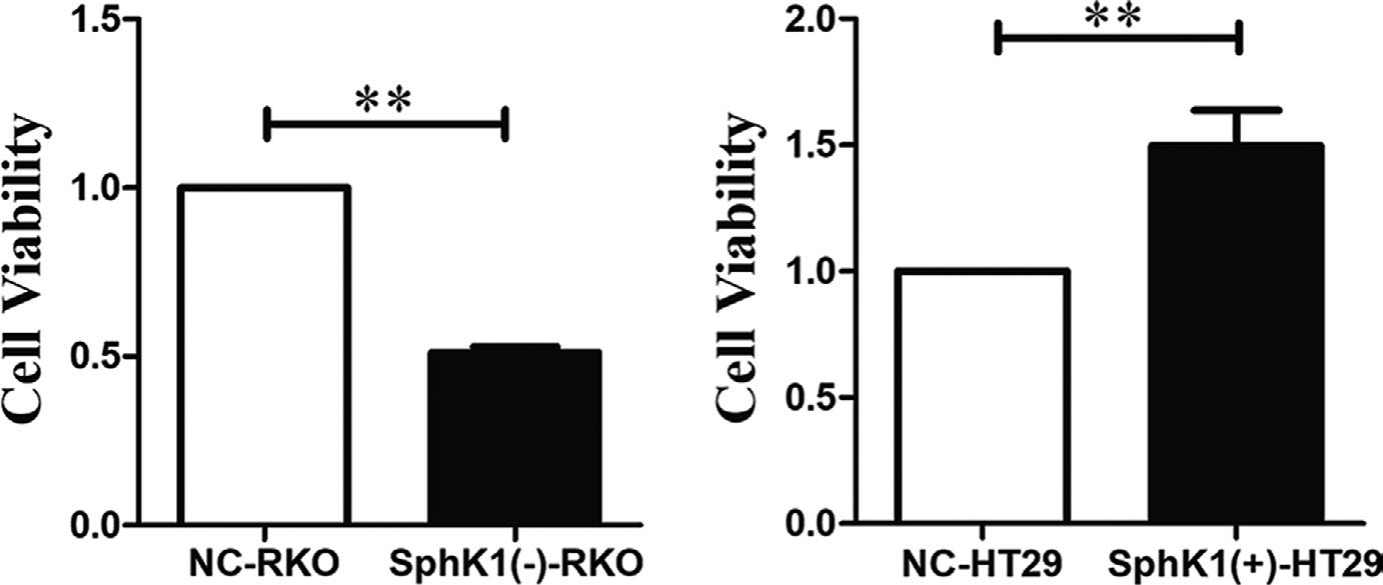
Overexpression of SphK1 significantly promoted viability of HT29 cells. CCK8 assay of CRC cell viability in vitro. (**p < 0.01 vs. NC‐RKO or NC‐HT29 group). Data are shown as the mean ± SD of three replicates.

CCK8 assay was used to detect the cell viability of RKO cells and HT29 cells. Compared with NC group, there was a marked increase in the cell viability of SphK1(+)‐HT29 cells and there was a marked decrease in the cell viability of SphK1(‐)‐RKO cells (Figure 3). It revealed that SphK1 promoted the growth of CRC cells in vitro.

### 3.4 SphK1 enhanced the migrational potency of CRC cells

**FIGURE 4:**
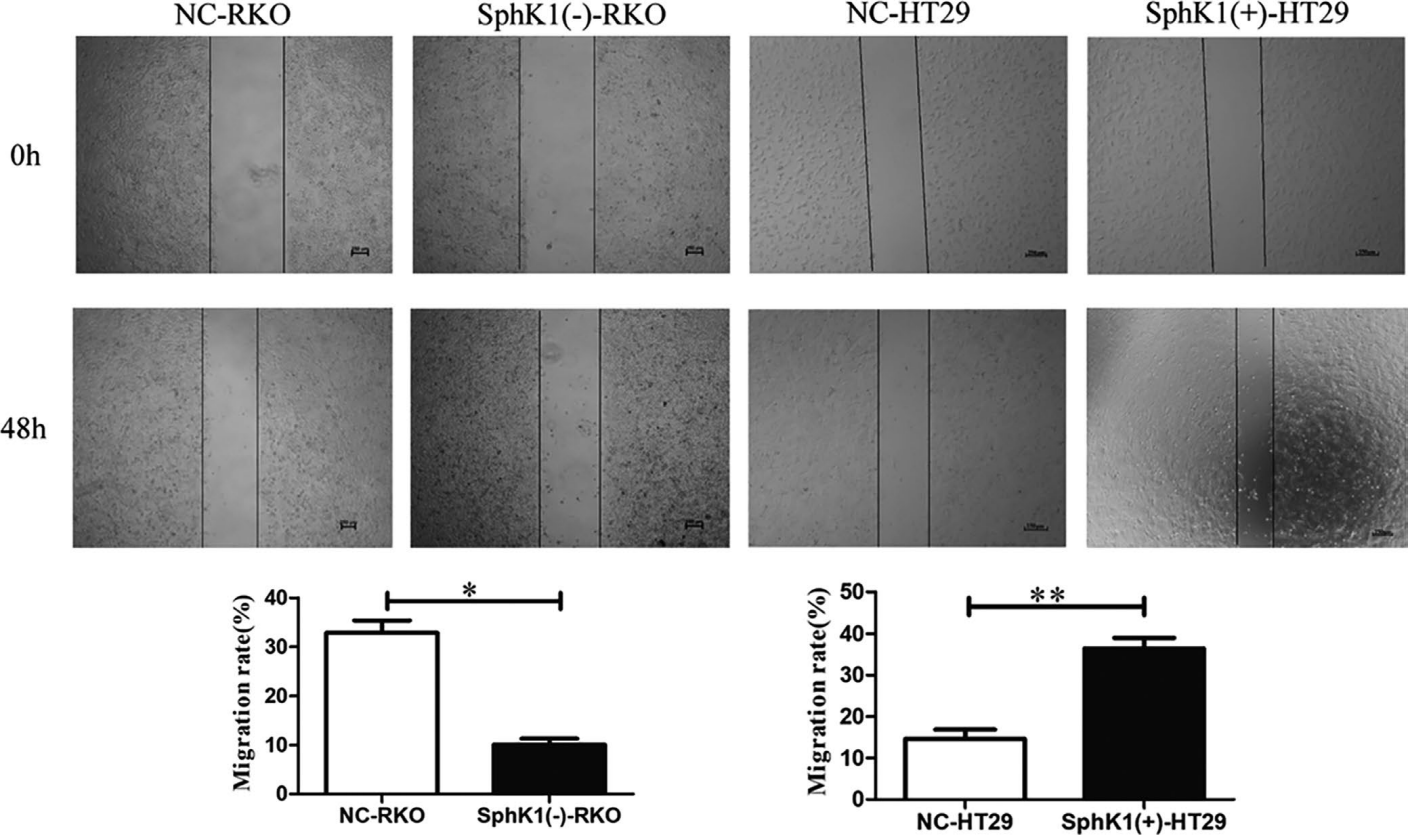
Overexpression of SphK1 promote migration and metastasis of CRC cells. Wound healing assay showing migrated RKO and HT29 cells transfected with shSphK1 or overexpression SphK1. (magnification, ×40, *p < 0.05 or **p < 0.01 vs. NC‐RKO or NC‐HT29 group). Data are shown as the mean ± SD of three replicates.

**FIGURE 5:**
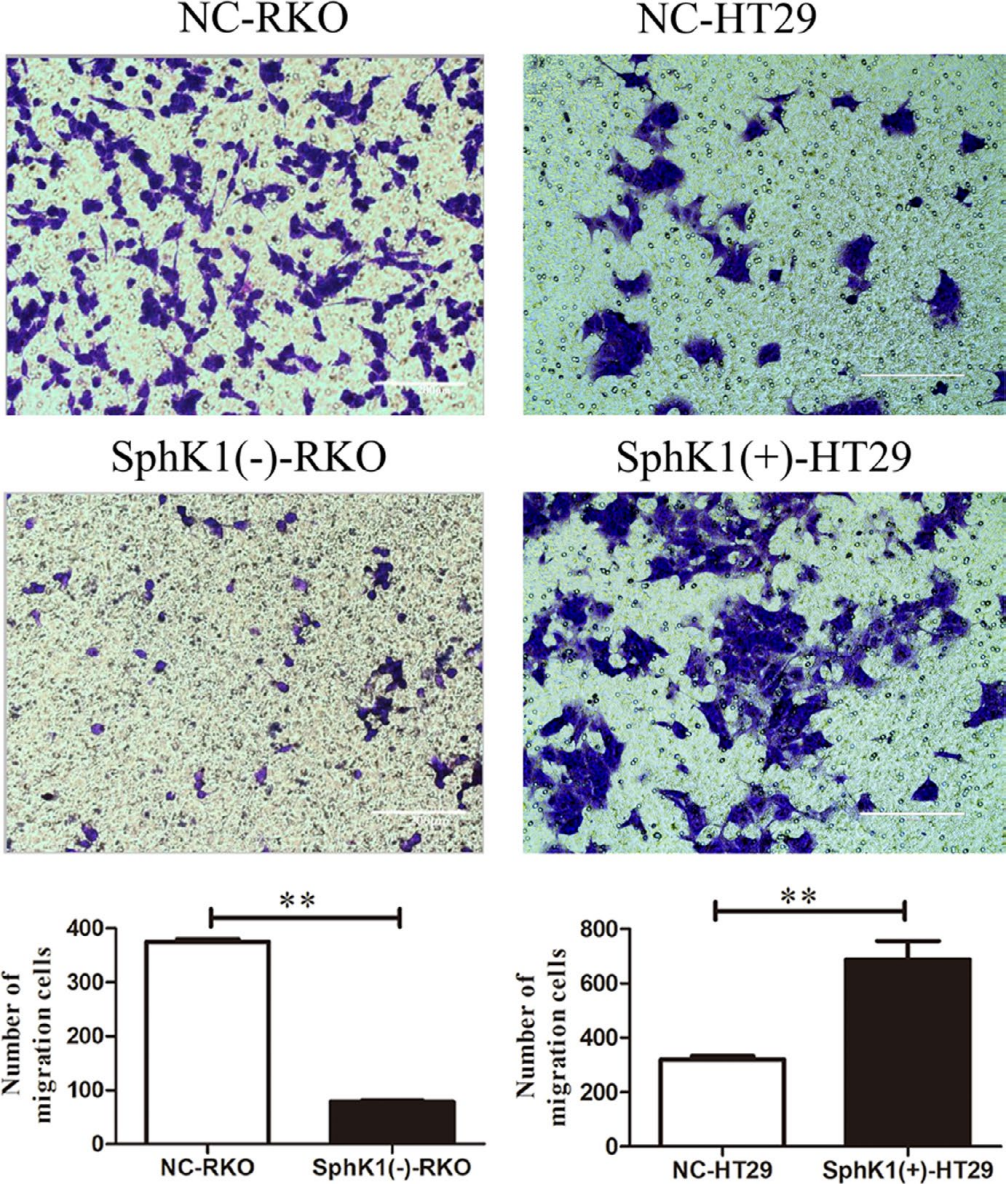
Overexpression of SphK1 promote migration and metastasis of CRC cells. Transwell assay of showing migrated RKO and HT29 cells transfected with shSphK1 or overexpression SphK1. (magnification, ×200, **p < 0.01 vs. NC‐RKO or NC‐HT29 group). Data are shown as the mean ± SD of three replicates.

The wound healing and transwell assays were used to detect the migrational potency of CRC cells. Compared with NC group, the number of migrated cells and wound healing rate were increased in SphK1(+)‐HT29 group, and the number of migrated cells and wound healing rate were decreased in SphK1(‐)‐RKO group (Figures 4 and 5). It revealed that SphK1 enhanced the migrational potency of CRC cells.

### 3.5 SphK1‐induced autophagic flux in CRC cells

**FIGURE 6:**
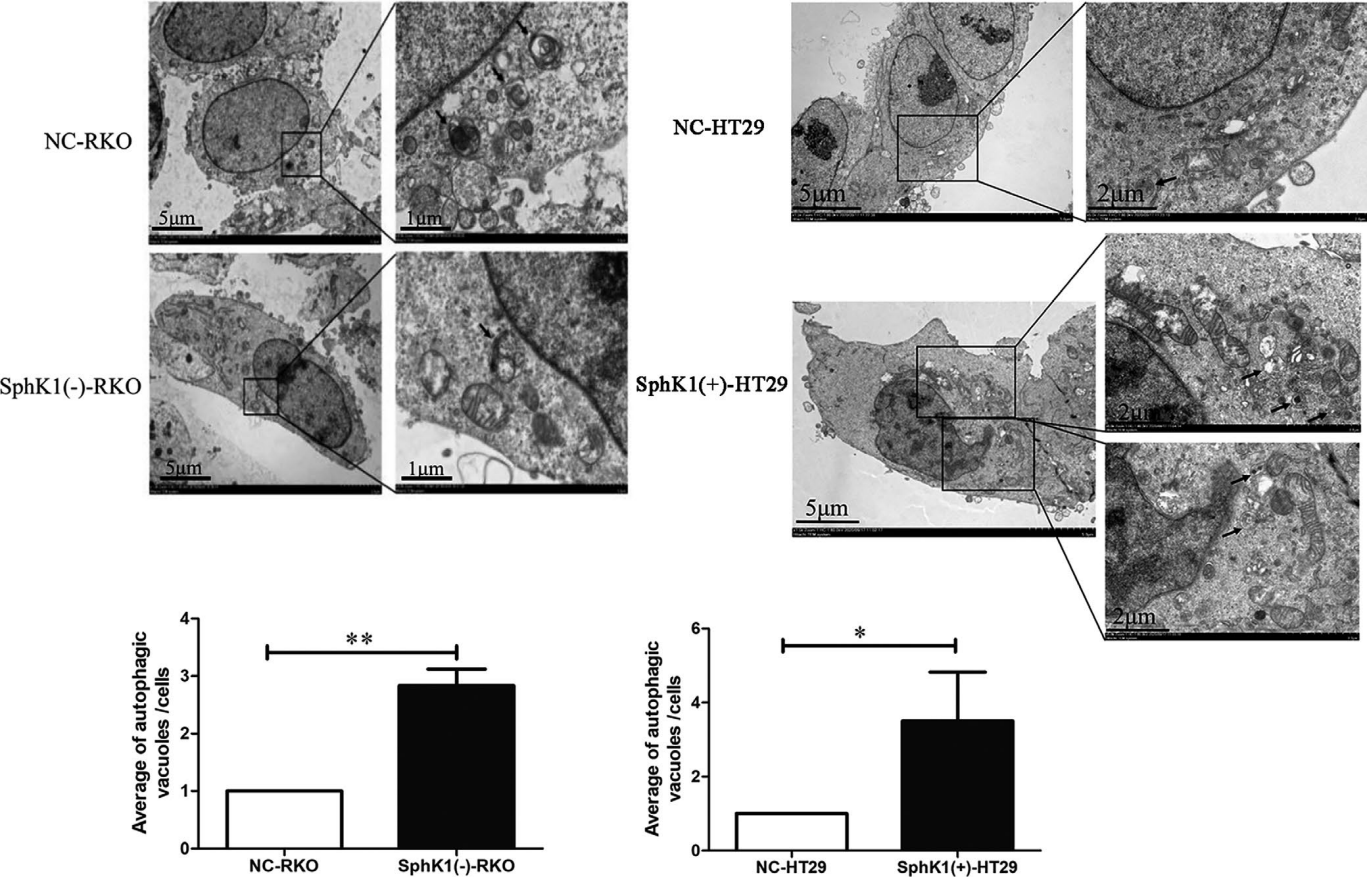
SphK1 is involved in induced autophagy in CRC cells. Transmission electron microscopy showing formation of autophagy flux after transfected with shSphK1 or overexpression SphK1 in RKO and HT29 cells. The arrow shows the autophagosomes/autolysosomes. (*p < 0.05, **p < 0.01 vs. NC‐RKO or NC‐HT29 group). Data are shown as the mean ± SD of three replicates.

The autophagosome/autolysosome in cells was detected by transmission electron microscopy. Compared with NC group cells, SphK1 knockdown inhibits autophagic flux in CRC cells, and overexpression of SphK1‐induced autophagic flux in CRC cells. (Figure 6).

### 3.6 SphK1 regulated the expression of protein related to autophagy, FA, and EMT

**FIGURE 7:**
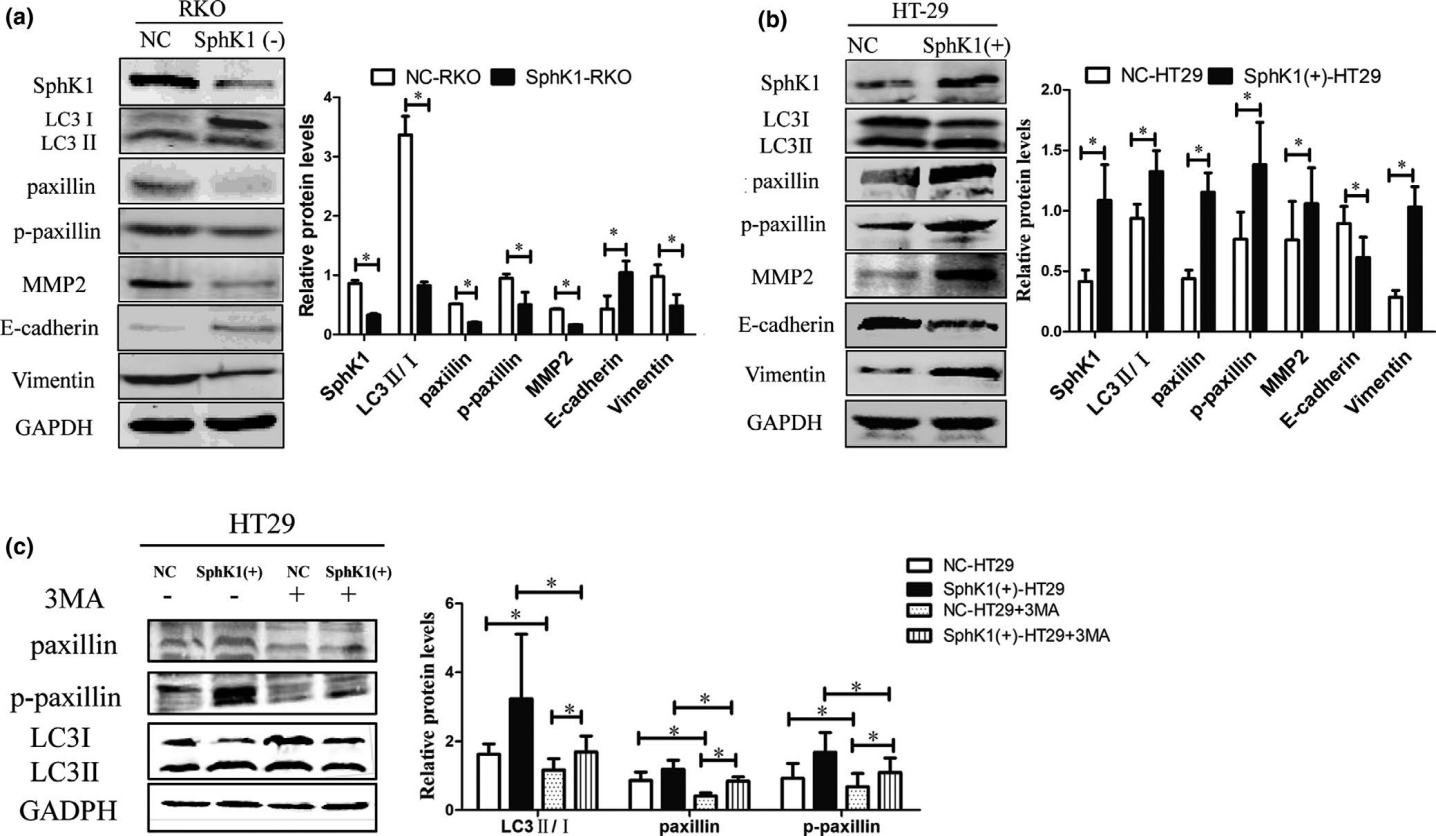
SphK1 facilitates autophagy, EMT factors, and focal adhesions pathway. (A, B) Western blot analysis of SphK1, LC3Ⅱ/Ⅰ, paxillin, p‐paxillin, MMP2, E‐cadherin, and vimentin in CRC cells. (*p < 0.05 vs. NC‐RKO group, *p < 0.05 vs. NC‐HT29. (C) Western blot revealing LC3, paxillin, and p‐paxillin after 3MA treatment (10 mM, 24 h) in SphK1(+)‐HT29. (*p < 0.05 vs. NC‐HT29 group, SphK1(+)‐HT29 group or NC‐HT29 group +3MA). Data are shown as the mean ± SD of three replicates.

As showed in the figures, the expression of paxillin, p‐paxillin, LC3Ⅱ/Ⅰ, vimentin, and MMP2 was enhanced and the expression of E‐cadherin was suppressed with the overexpression of SphK1 in HT29. In contrast, SphK1 knockdown could inhibit the expression of paxillin, p‐paxillin, LC3Ⅱ/Ⅰ, vimentin, and MMP2 and enhance the expression of E‐cadherin in RKO cells. Moreover, the expression of paxillin and p‐paxillin can be inhibited significantly by the autophagy inhibitors 3‐MA in SphK1(+)‐HT29 cells (Figure 7).

### 3.7 SphK1 knockdown inhibited the growth of orthotopic implantation tumor and affected the expression of LC3, paxillin, and p‐paxillin in orthotopic tumor tissues

**FIGURE 8:**
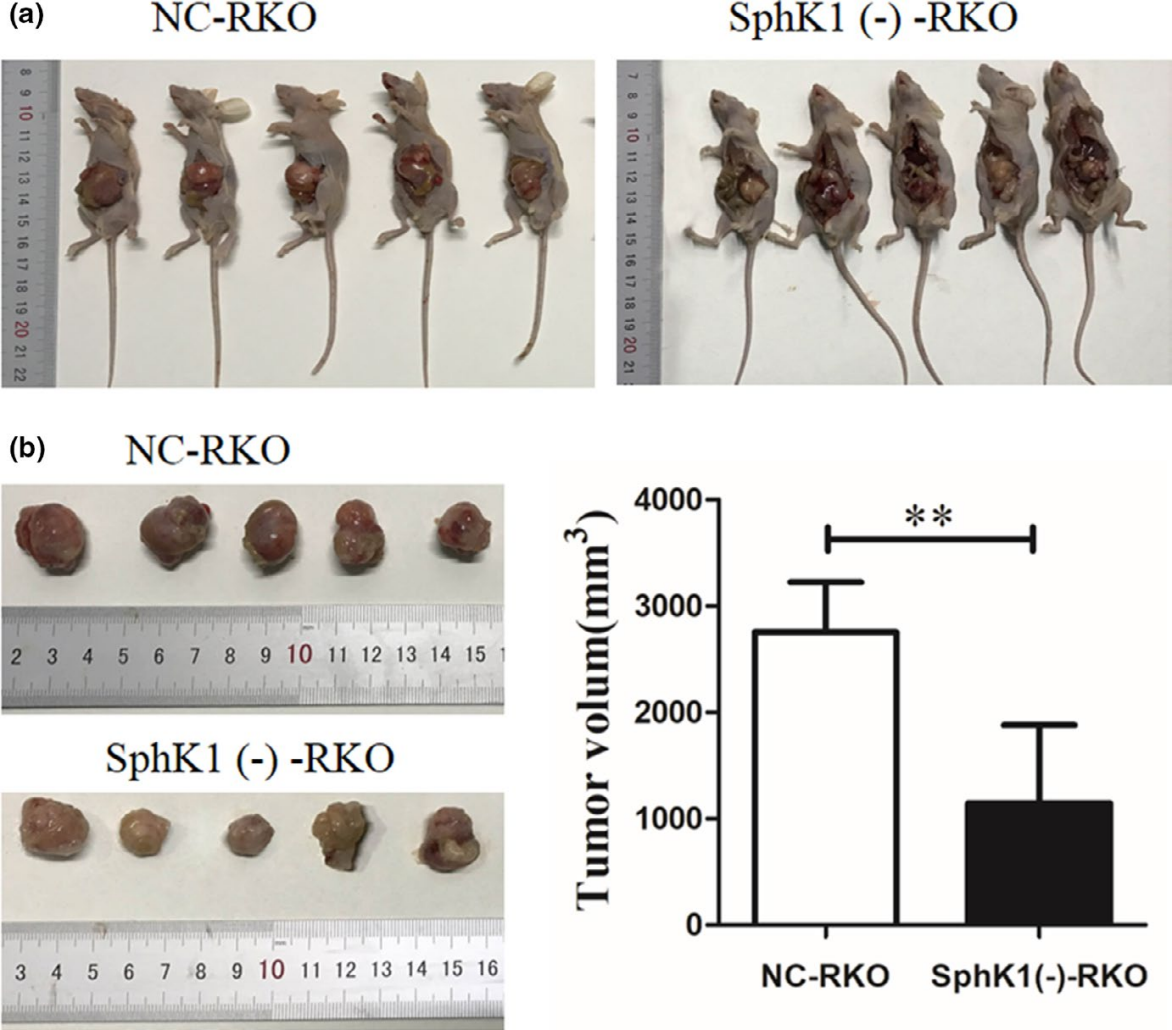
The silencing of SphK1 in RKO cells leads to delayed growth of tumor in nude mice. (A) The orthotopic transplantation nude mice in SphK1(‐)‐RKO and NC‐RKO groups. (B) The size of orthotopic tumors in transplanted nude mice in SphK1(‐)‐RKO and NC‐RKO groups. (**p < 0.01 vs. NC‐RKO group). Data are presented as mean ± SD (*n* = 5).

**FIGURE 9:**
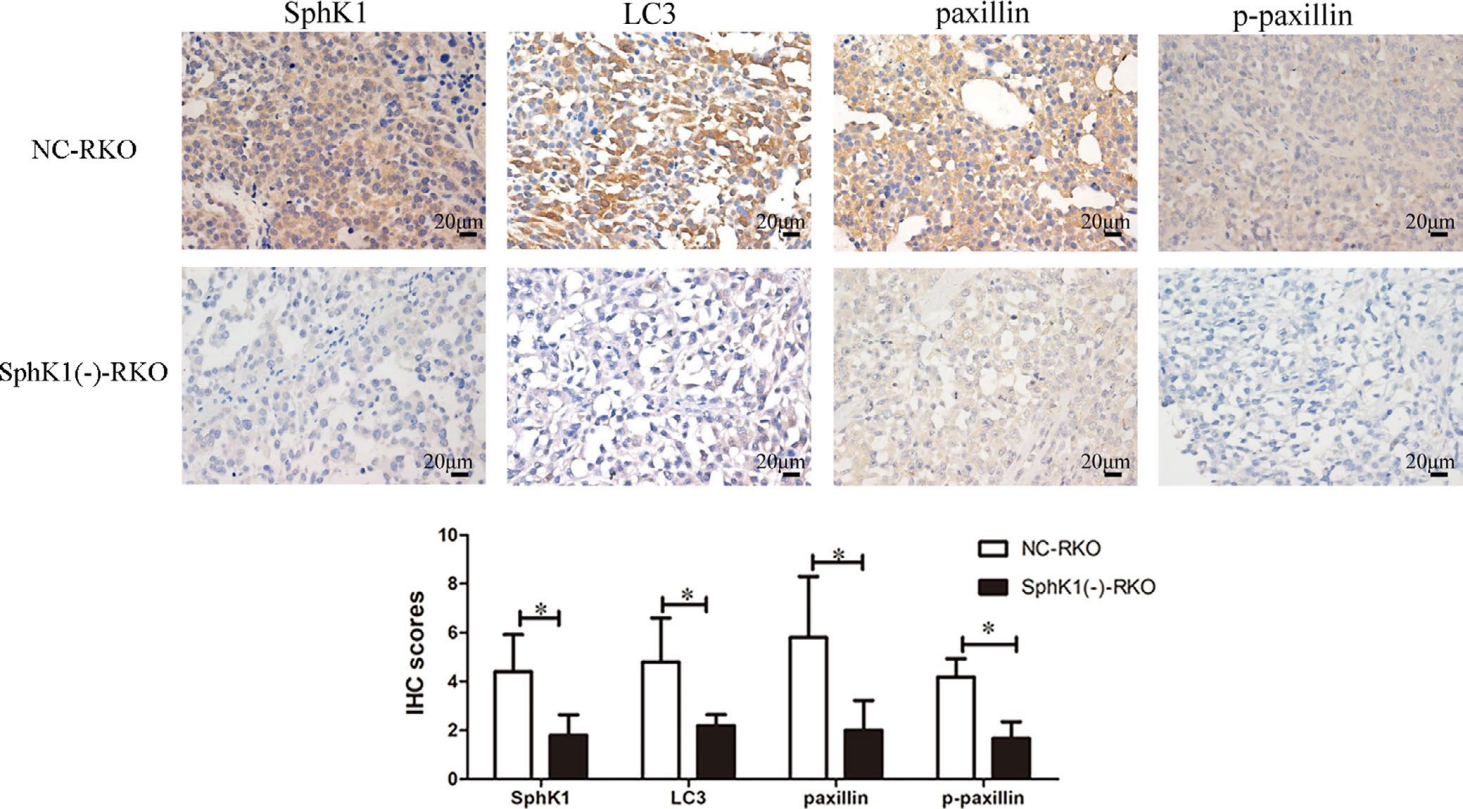
Low expression of SphK1, LC3, paxillin, and p‐paxillin in SphK1(‐)‐RKO. Immunohistochemical staining of SphK1, paxillin, p‐paxillin, and LC3 in orthotopic tumor tissues. (magnification, ×400, *p < 0.05 vs. NC‐RKO group). Data are shown as the mean ± SD of three replicates.

**FIGURE 10:**
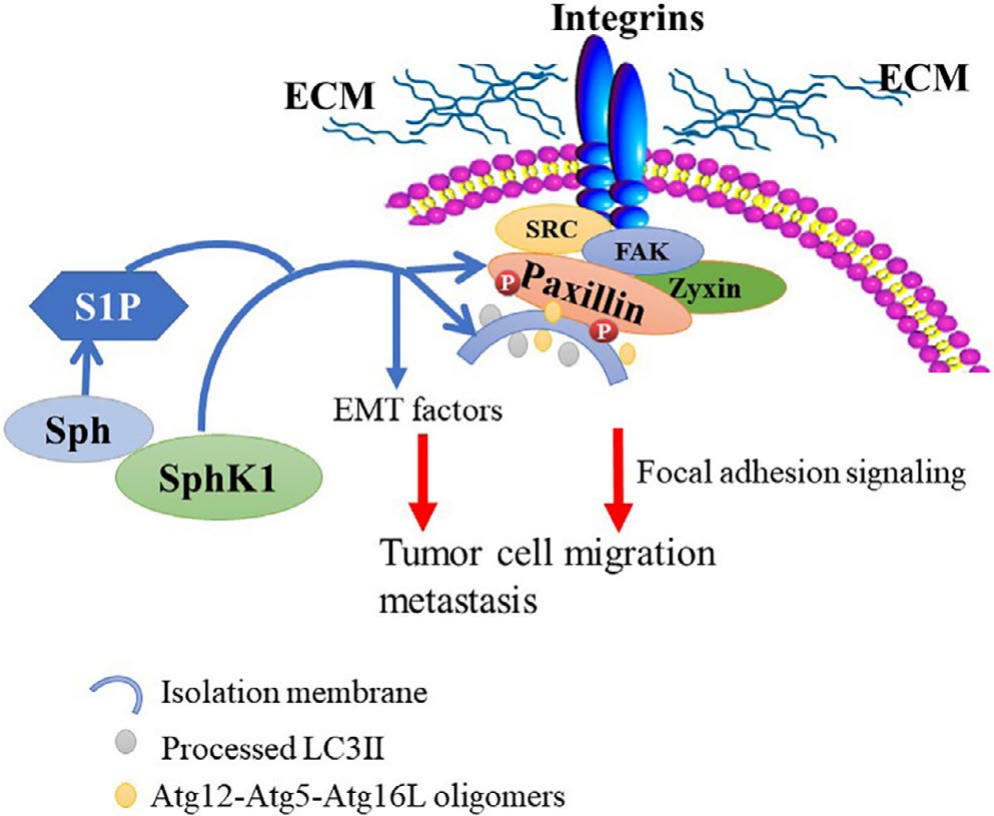
Schematic model showing the role of SphK1 in the regulation of EMT progression and FA signaling.

The tumors volumes of SphK1(‐)‐RKO group were smaller than those of NC‐RKO group in colonic orthotopic implantation model (Figure 8). Moreover, with the suppression of SphK1, the expression of LC3, paxillin, and p‐paxillin was decreased in tissues of orthotopic tumors (Figure 9). The schematic model of this study was shown in Figure 10.

## 4 DISCUSSION

Due to the absence of typical symptoms in the early stage of CRC, most patients are under advanced stage when they are diagnosed. Invasion and metastasis become the major cause of cancer‐related death. Hence, investigation of the metastasis mechanism is critical to improve the diagnosis and treatment of CRC.

The formation and disassembly of adhesions are irreplaceable of cell migration. Paxillin, an adhesion adaptor protein that plays an important role in regulating adhesion formation and turnover.^12^, ^19^ In present study, it was demonstrated that SphK1‐induced autophagy expression and facilitated paxillin and its phosphorylation. Moreover, SphK1‐driven autophagy was required for paxillin and its phosphorylation‐mediated invasion and metastasis of CRC cells.

Autophagy is a biological phenomenon mediated by lysosomes to degrade damaged proteins or organelles in cells, it is an important process to maintain the homeostasis of intracellular environment to adapt to environmental changes.^20^ Several studies have demonstrated that autophagy is involved in the occurrence and development of tumors.^21^ Moreover, autophagy promoted cancer cells migration and invasion.^22^ Autophagy as a highly complex mechanism of tumorigenesis was regulated by multiple signal pathways.^23^ Previous studies showed that SphK1 is involved in autophagy, and it showed that the inhibition of SphK1 impaired the fusion of endosomal membranes trafficking to dysfunctional enlarged late endosomes, and reduced autophagy flux.^24^

As a lipid kinase that phosphorylate sphingosine to S1P, SphK1 involves many physiological and pathophysiological processes, but are especially active in cancer cells where they can promote proliferation, migration, invasion, and metastasis.^25^, ^26^ High expression of SphK1 was detected in several tissues and cells,^27^ the study showed that SphK1 promoted the migration and metastasis of CRC via inducing EMT which was mediated by the FAK/AKT/MMPs axis.^28^ As an important phosphorylation substrate of focal adhesion kinase (FAK)/Src kinase, paxillin, and its phosphorylation can promote the dynamic and assembly of FA, and further promote cell movement, invasion, and metastasis.^29^ Recently study showed norcantharidin adjusted the migration and invasion abilities of YD‐15 cell regulating the FAK/paxillin axis.^30^ And MLK3 promoted migration and invasion of breast cancer cells through regulating the phosphorylation of paxillin.^31^ In the present study, the expression of SphK1 and paxillin enhanced in CRC of patients with lymphatic and distant metastases. Further study showed that SphK1 not only regulate the invasion and migratory, but also regulate the expression of paxillin and its phosphorylation in CRC cells in vitro and vivo. Previous study showed paxillin phosphorylation promoted adhesion between cancer cells and matrix, inhibited adhesion between cells, enhanced pseudopodia formation, invasion, and metastasis of cancer cells.^14^ Moreover, phosphorylated paxillin is closely related to poor prognosis of patients with mobile tongue squamous cell carcinoma.^32^ Hence, SphK1 may potentiate focal adhesion paxillin and its phosphorylation‐mediated metastasis in CRC. However, the mechanism of invasion and metastasis regulated by SphK1 and paxillin in CRC is still unclear.

The study showed that autophagy and paxillin had genetic interactions was the first found in Drosophila melanogaster.^33^ Previous research showed that autophagy promoted the expression of paxillin and it leaded to enhance migration of HeLa cells.^34^ Additionally, loss of ATG5 function made the expression of paxillin reduced, resulting in stable adhesion plaques and reduced directed cell motility, and autophagy enhanced FAK signaling and degraded paxillin to promote FA disassembly to suppress cell adhesion then resulted in less cell spreading.^35^ The present study demonstrated that inhibition of paxillin was found in the SphK1 knockdown CRC cells in vitro and vivo, moreover, suppression of autophagy by 3MA reversed the expression of paxillin and its phosphorylation in SphK1‐overexpressed CRC cells. SphK1‐driven autophagy may induce the assembly and disassembly of FA via regulating paxillin and its phosphorylation. What an interesting thing is that study had showed that paxillin, which served as a crucial scaffolding and signal integrator, bound directly to microtubule‐associated protein 1 light chain 3 (LC3) through a conserved LC3‐interacting region (LIR) motif to stimulate FA disassembly and promote tumor cell metastasis.^36^ LC3 is a key to evaluate the levels of autophagy in cells.^37^, ^38^ Binding to LC3 is an important criterion for identifying autophagy regulatory proteins in cells, autophagy‐related proteins contain a LIR by interacting with LC3 to send substrates to lysosomes for digestion and degradation.^39^ SphK1‐driven autophagy may promote CRC metastasis via mediating the direct interacting of paxillin and LC3.

Cell migration is a complex process that requires the continuous, coordinated formation, and disassembly of adhesions, paxillin played an important role in this process. These studies highlight that SphK1‐driven autophagy may potentiate focal adhesion paxillin‐mediated metastasis in CRC. However, the detailed mechanism among SphK1, paxillin, and autophagy in invasion of CRC still need further research.

## ETHICS APPROVAL AND CONSENT TO PARTICIPATE

This study was approved by Medical Ethics Committee of The First Affiliated Hospital of Guangxi Medical University (Guangxi, China), and written informed consent was obtained from all patients. All animal experiments complied with the Policy of Guangxi Medical University on the Care and Use of Laboratory Animals.

## CONFLICT OF INTEREST

The authors declare that they have no competing interests.

## AUTHOR CONTRIBUTIONS

Jiangni Wu, Lan Lin, and Shiquan Liu developed the original hypothesis and supervised the experimental design; Jiangni Wu, Lan Lin, Shibo Luo, Liye Zhu, and Zhenhua Fu performed in vitro and in vivo experiments; Mengbin Qin, Zhihai Liang, and Jiean Huang participated in the clinical specimens collection; Jiangni Wu, Xinze Qiu, Da Chen, and Erdan Wei analyzed the data and performed statistical analysis; Jiangni Wu, Lan Lin, and Shiquan Liu wrote and revised the manuscript. All authors read and approved the final manuscript.

## Data Availability

The datasets used and/or analyzed during the current study are available upon reasonable request.
